# Proteomic Profiling of Pre- and Post-Surgery Saliva of Glioblastoma Patients II: A Preliminary Investigation of the Complementary Low Molecular Mass Fraction

**DOI:** 10.3390/ijms26209995

**Published:** 2025-10-14

**Authors:** Alexandra Muntiu, Federica Vincenzoni, Diana Valeria Rossetti, Massimo Castagnola, Irene Messana, Federica Iavarone, Andrea Urbani, Giuseppe La Rocca, Alessio Albanese, Alessandro Olivi, Giovanni Sabatino, Claudia Desiderio

**Affiliations:** 1 Dipartimento di Scienze Biotecnologiche di Base, Cliniche Intensivologiche e Perioperatorie, Università Cattolica del Sacro Cuore, 00168 Rome, Italy; 2 Fondazione Policlinico Universitario A. Gemelli IRCCS, Università Cattolica del Sacro Cuore, 00168 Rome, Italy; 3 Istituto di Scienze e Tecnologie Chimiche “Giulio Natta”, Consiglio Nazionale delle Ricerche, 00168 Rome, Italy; 4 Centro Europeo di Ricerca sul Cervello-IRCCS Fondazione Santa Lucia, 00179 Rome, Italy; 5 Institute of Neurosurgery, Fondazione Policlinico Universitario A. Gemelli IRCCS, Università Cattolica del Sacro Cuore, 00168 Rome, Italy

**Keywords:** peptidomics, biomarkers, saliva, liquid biopsy, brain tumor, glioblastoma, mass spectrometry, top–down proteomics

## Abstract

This research aimed to analyze the proteomic profile of the low-molecular mass fraction of salivary pools from patients with glioblastoma IDH wild type (GBM) to disclose the small protein and peptide components, including protein fragments, cryptides, and tumor-associated peptides, still lacking specific information in the literature, to the best of our knowledge. This fraction, corresponding to the unretained proteome fraction, was obtained by pretreating the acid-soluble fraction of saliva through Filter-Aided Sample Preparation devices with a filter molecular cutoff of 10 kDa. The fraction was analyzed by LC-MS in its entire form, without trypsin pre-digestion, following a top–down approach. Data from the analysis of pre- and post-operative salivary pools from patients with newly diagnosed and recurrent GBM were compared and discussed with data obtained in our previous study on the complementary salivary proteome fraction > 10 kDa analyzed by a bottom–up approach and data from the literature. The results highlighted a panel of GBM-associated peptide fragments from different protein precursors, namely, ANXA1, CFL1, GLUL, PFN1, H2AC12, ACTB, and HBB, which are suggested for further exploration as potential diagnostic and prognostic biomarkers and clinical applications. These findings, although providing only preliminary results on a small scale, offer new insights into the molecular characteristics of GBM tumor and lay the groundwork for further investigations on a large scale using saliva liquid biopsy for biomarker discovery and validation. The aim is to advance precision medicine and improve clinical outcomes in GBM, one of the most aggressive brain tumors with a poor prognosis, for which early diagnosis and monitoring of treatment response remain significant challenges.

## 1. Introduction

Glioblastoma (GBM) IDH-wild type is the most frequent and prevalent tumor of the Central Nervous System (CNS) in adults and is classified as World Health Organization (WHO) grade 4 [1,2]. GBM is a highly aggressive cancer with an unfavorable outcome [3], which is very difficult to treat; indeed, the median survival is about 15 months for patients who receive standard therapy (surgical resection followed by co-adjuvant radio-chemotherapy) and less than 1 year in population-based studies [4]. The tumor, marked by rapid growth and the capability to invade and infiltrate surrounding healthy brain tissue, poses a challenge to its total surgical resection [5]. In addition, a small subpopulation of GBM cancer stem cells (GSC) contributes, thanks to their pluripotent and self-renewing capabilities, to tumor chemo- and radiotherapy resistance [6], leading to recurrence [7]. The incidence of GBM is two to five cases per 100,000 people per year in the USA and Europe [4]. The diagnosis of this tumor in the early stages can significantly influence the patient’s prognosis, therapeutic approach, comorbidities, survival expectations, and recurrence rate [8]. In recent years, the search for non-invasive diagnostic methodologies, cheap and accurate, has proved to be crucial. Tissue biopsies, currently and traditionally used, invasive for the patient despite being informative, are not proper for early diagnosis and/or the monitoring during the follow-up and the ongoing treatments, resulting in the need to identify biomarkers in liquid biopsies from the perspective of a precision medicine approach. Early diagnosis of cancer is essential for improving survival rates, allowing for the development of appropriate and less aggressive treatments that impact the patient’s quality of life, since cancer is often more treatable in the early stage [9]. From this standpoint, saliva emerged as a source of biomarkers for early detection of cancer as a result of multi-omics investigations [9], representing an attractive diagnostic fluid collected non-invasively, easy to store, and inexpensive when compared to other bodily fluids utilized in clinical laboratories [10,11]. Indeed, liquid biopsy, including saliva, can be predictive for the treatment and prognosis of cancers, including gliomas, as it can provide early perceptions into disease progression [12,13]. In saliva, proteins, peptides, and other factors from salivary glands are enriched with blood filtrate [14]; however, saliva, compared to other biofluids such as plasma or serum, shows the advantage of a lower content of abundant proteins, which makes it easier to identify low-abundance circulating biomarkers. This reduces complex manipulations before analysis, saving time, costs, and reagents. The discovery of potential biomarkers in saliva may have application in early diagnosis of disease, when the tumor is still small, more responsive to treatment, and not yet metastasized [15], or in the monitoring of recurrence during patient follow-up. In recent years, mass spectrometry proteomics has been widely applied to biomarker discovery [16,17], and, specifically, to GBM profiling in our previous investigations on tumor aspirate fluid [18,19,20] and saliva [21].

The present investigation aimed at characterizing and comparing the proteomic profile of the low molecular mass fraction (<10 kDa) of GBM patients’ saliva pooled per tumor type, newly diagnosed (ND) and relapsed (R) glioblastoma, and time of collection, i.e., T0 (pre-surgery saliva), T1 and T3 (1 month and 3 months post-surgery saliva, respectively), analyzed by LC-MS in the intact form without pre-enzymatic digestion (top–down approach). The proteomic analysis of the low molecular mass fraction of the salivary proteome specifically aims to identify small proteins, peptides, and protein fragments that may characterize GBM saliva and which could include cryptides [22], tumor-associated peptides [23], and long non-coding RNA peptides [24,25], challenging topics in current research. The resulting data of this investigation are complementary to data obtained on the proteome fraction > 10 kDa of the same sample pools after trypsin digestion [21], accomplishing a preliminary comprehensive molecular overview of GBM saliva by top–down/bottom–up integrated proteomic platforms using Filter-Aided Sample Preparation (FASP) pretreatment. In fact, while the bottom–up approach was applied to the proteomic analysis of the high molecular mass fraction retained by the 10 kDa FASP filter device [21], the low molecular mass fraction < 10 kDa, representing the corresponding unretained filtrate, generally discarded after the first centrifugation, was analyzed in the present study by a top–down approach to investigate the low molecular mass proteome fraction of GBM saliva, never explored.

Currently, the study of the salivary peptidome in GBM patients remains a little-explored area of research, but it presents significant potential. Indeed, tumors release several factors into the bloodstream or other biofluids that are able to cross the Blood–Brain Barrier (BBB), including microRNAs, circulating tumor DNA and tumor cells, proteins, extracellular vesicles (exosomes), and metabolites, opening the prospect of screening glioblastoma with minimally invasive analysis and multi-omics sciences profiling [13]. Peptidomic profiling could, therefore, lead to the identification of novel biomarkers for disease diagnosis and monitoring of the tumor in the early stage and/or of the relapse, while also providing deeper insights into the molecular mechanisms underlying the disease. The comparison of data from analysis of saliva with those obtained previously from GBM tumor aspirate fluid [18,20] consolidates the results of this pilot investigation involving different biological matrices. The study of peptidomics in cancer is intriguing for the exploration of new diagnostic tools and clinical applications, as well as for the development of targeted molecular therapies, in which the low side effects of peptides are challenging compared to conventional cancer treatments [5]. The data presented, considering the investigation on a small scale, and the supporting literature, outline potential GBM salivary biomarkers to deeply explore and validate in future studies, already providing a valuable reference for ongoing glioblastoma research.

## 2. Results and Discussion

The profiling of the low molecular mass fraction of the salivary proteome, the focus of the present study, poses a significant challenge for the initial characterization of the small protein and peptide components associated with GBM disease, which still lack specific investigations, to the best of our knowledge. This preliminary research enrolled approximately twenty GBM patients, but it was possible to collect all types of biological samples under investigation from only six patients. The biological samples were then pooled based on tumor type, ND and R GBM, and time of collection, pre- and post-surgery, to reduce the inter-individual variability and perform a first overview of the proteomic profiles differences by LC-MS analysis in triplicate runs. These results complement previous data obtained by analysis on the proteome fraction > 10 kDa of the same saliva pools analyzed by bottom–up and FASP digestion [21] and were obtained following the same approach previously applied to GBM aspirate fluid [20]. The present results thus finalize a pilot comprehensive exploration of the salivary proteome of the same sample pools in a wide range of molecular mass.

Specifically, saliva samples were collected at different time points, i.e., from ND GBM patients before surgery (NDT0), 1 and 3 months post-operative (NDT1 and NDT3), and from patients with R GBM before the tumor removal (RT0), defining five groups of saliva pools, including a pool of healthy controls (CTRL).

To ensure confident results, stringent filters were applied to LC-MS data elaboration to select exclusively the unique peptides identified with high confidence and repeatability in all analytical replicates, with a particular focus on identifying key disease-associated peptides. This approach was already employed in our previous study on GBM CUSA fluid peptidome [20]. Although this procedure significantly reduced the number of identifications, it ensured reliable analytical data for a pilot exploration and feasibility study for biomarker discovery.

**Figure 1:**
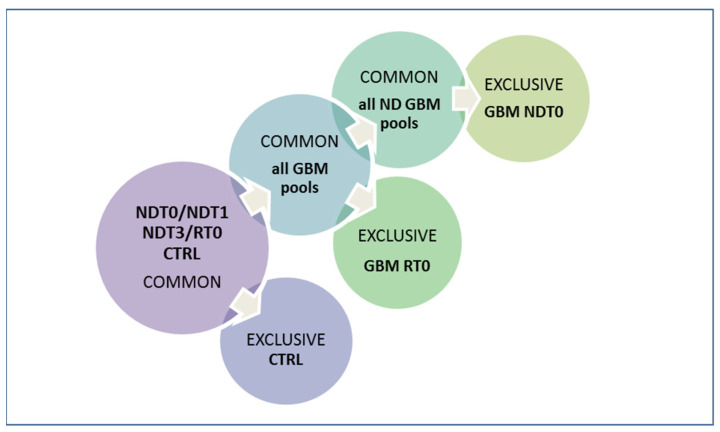
Workflow of saliva liquid biopsy peptidomics data analysis and elaboration. The identifications common to GBM and CTRL saliva pools were compared for relative quantitation and comparative analysis of peptide feature distribution. Identifications exclusive of GBM pools with respect to CTRL and of ND GBM versus R GBM pre-surgery saliva were compared for pilot exploration of potential diagnostic and prognostic disease biomarkers.

The obtained proteomic data were analyzed by grouping analysis [26] following the workflow shown in Figure 1, in order to identify, at first, the peptides common to all GBM samples and CTRL, and then evaluate their quantitative differences among the pools. Peptides peculiar to all pathological specimens were analyzed for their distribution in subgroups, also distinguishing the elements exclusive of the ND and R T0 samples to highlight potential biomarkers of disease and recurrence.

**Figure 2:**
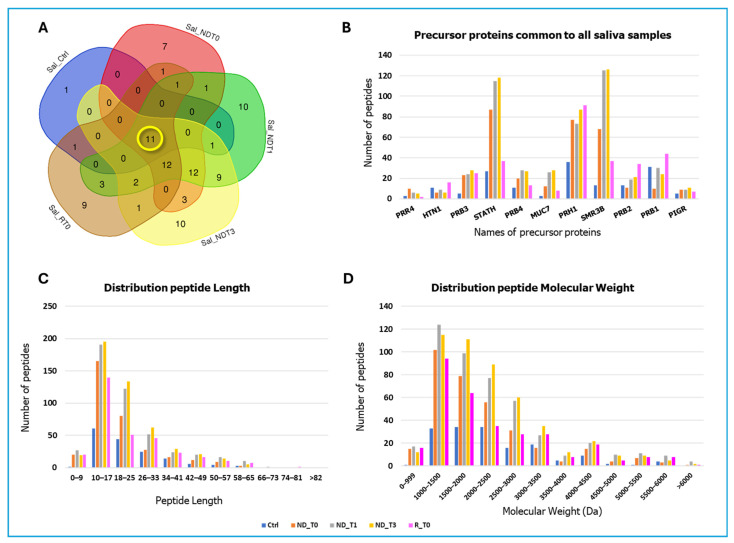
Data on the peptides and relative precursor proteins obtained by LC-MS analysis of NDT0, NDT1, NDT3, RT0 GBM, and CTRL saliva pools. (**A**) Venn diagram resulting from grouping analysis of the protein precursors of the peptides identified in all pools, highlighting 11 precursor proteins common to all saliva samples, marked in yellow. (**B**) Bar graph illustrates the number of unique peptides identified for each of the 11 shared precursor proteins, with quantitative differences highlighted across the analyzed groups: CTRL (blue), NDT0 (orange), NDT1 (grey), NDT3 (yellow), and RT0 (pink). (**C**) Bar graph illustrates the distribution of the number of amino acid residues (peptide length) of the identified peptides across all pools. (**D**) Bar graph illustrates the distribution of molecular weight (Da) of the identified peptides across all pools.

A total of 507, 629, 702, 380, and 161 peptide sequences, originating from 48, 62, 61, 41, and 14 precursor proteins, were identified in NDT0, T1, T3, RT0, and CTRL saliva, respectively (complete identification data in Supplementary Tables S1–S5). To compare pathological samples with controls, a grouping analysis of the precursor proteins identified in the five saliva pools was performed. The resulting Venn diagram revealed 11 precursor proteins shared by all the samples (Figure 2A), namely, the Proline-rich protein 4 (PRR4), Histatin-1 (HTN1), Basic salivary proline-rich proteins 1, 2, 3, and 4 (PRB1, PRB2, PRB3, PRB4), Statherin (STATH), Mucin-7 (MUC7), Salivary acidic proline-rich phosphoprotein 1/2 (PRH1), Submaxillary gland androgen-regulated protein 3B (SMR3B) (also called Proline-rich peptide P-B), and Polymeric immunoglobulin receptor (PIGR). According to The Human Protein Atlas [27], these proteins are classified as highly expressed in the salivary glands. Figure 2B presents a bar graph displaying the number of unique peptides identified for each of these 11 proteins, providing a quantitative comparison across the different pools. Notably, for the majority of the 11 precursor proteins analyzed, a greater number of peptides were identified in GBM saliva samples compared to CTRL. However, specific exceptions were noted: for the precursor proteins HTN1 and PRB1, the number of peptides identified in the CTRL pool was higher than that detected in all the ND GBM pools, except for the RT0 group. The same finding was observed for PRB2 levels in CTRL, specifically with respect to NDT0. The graph in Figure 2C illustrates the comparative distribution of peptide lengths among the different groups. The peptide lengths were found predominantly in the ranges of 10–17 to 18–25 amino acid residues. Of note, shorter peptides, less than 10 amino acids, were particularly identified in GBM samples compared to CTRL. Longer peptides, greater than 33 amino acids, were less commonly identified; however, different distributions between the pools have been observed, again highlighting the variations in biological processes and enzymatic activities under different conditions studied. The molecular mass of the majority of the identified peptides ranged from 1000 to 3000 Da (see Figure 2D).

**Figure 3:**
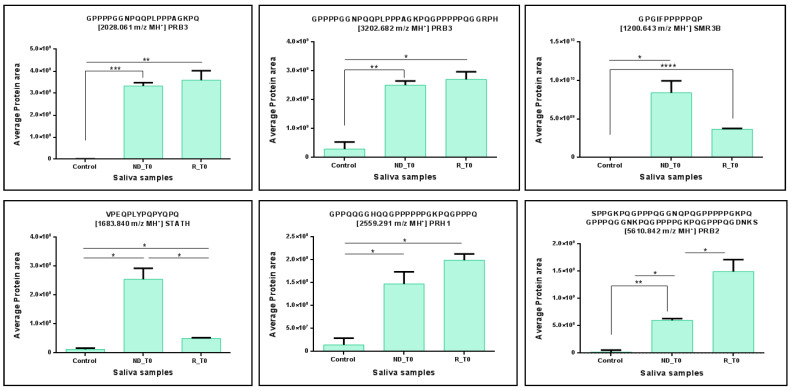
Bar chart of the label-free relative quantitation of peptide fragments of PRB2, PRB3, SMR3B, STATH, and PRH1 precursor proteins showing statistically significant difference between the CTRL and GBM NDT0 and RT0 pools. Significant differences were determined by one-way ANOVA and Tukey’s post-hoc test (* *p*-value < 0.05, ** *p*-value < 0.01, *** *p*-value < 0.001, **** *p*-value < 0.0001).

**Figure 4:**
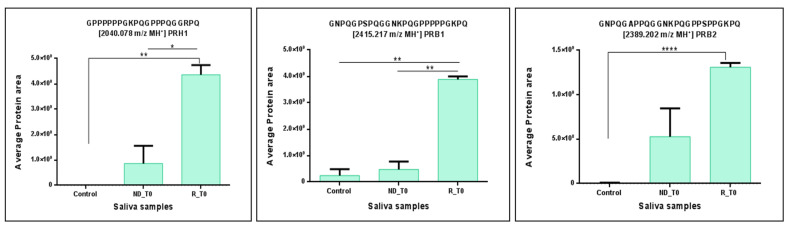
Bar chart of the label-free relative quantitation of peptide fragments of PRB1, PRB2, and PRH1 precursor proteins, showing statistically significant difference between CTRL and RT0 GBM pool. Significant differences were determined by one-way ANOVA and Tukey’s post-hoc test (* *p*-value < 0.05, ** *p*-value < 0.01, **** *p*-value < 0.0001).

From a quantitative standpoint, the statistical analysis performed using the ANOVA test revealed significant variations in some peptide fragments of the 11 precursor proteins analyzed between NDT0 and RT0 pools with respect to CTRL. Specifically, interesting and statistically significant quantitative variations were observed for peptides of PRB3 (2028.061 and 3202.682 *m*/*z* MH^+^), STATH (1683.840 *m*/*z* MH^+^), PRH1 (2559.291 *m*/*z* MH^+^), SMR3B (1200.643 and 1315.723 *m*/*z* MH^+^), and PRB2 (5610.842 *m*/*z* MH^+^). All these peptides showed significantly higher levels in ND and R GBM T0 saliva with respect to CTRL (Figure 3), resulting in a potential panel of peptides to monitor in saliva. Furthermore, of note are the significantly higher levels of the peptides of PRH1 (2040.078 *m*/*z* MH^+^), PRB1 (2415.217 *m*/*z* MH^+^), and PRB2 (2389.202 *m*/*z* MH^+^) in RT0 saliva, with respect to CTRL (Figure 4), a potential panel of peptides for early detection of tumor recurrence in the follow-up.

On the contrary, the peptide fragment SHREFPFYGDYGS (1561.672 *m*/*z* MH^+^) of HTN1 was found to mark CTRL saliva, although another fragment of the same protein with sequence YGDYGSNYLYDN was, however, found ubiquitous in the pools. Notably, according to previous studies [28,29], the peptide SHREFPFYGDYGS (1561.672 *m*/*z* MH^+^) was reported to exhibit a significant biological activity, particularly in wound healing. Its absence in saliva could be a feature of the pathological saliva.

**Figure 5:**
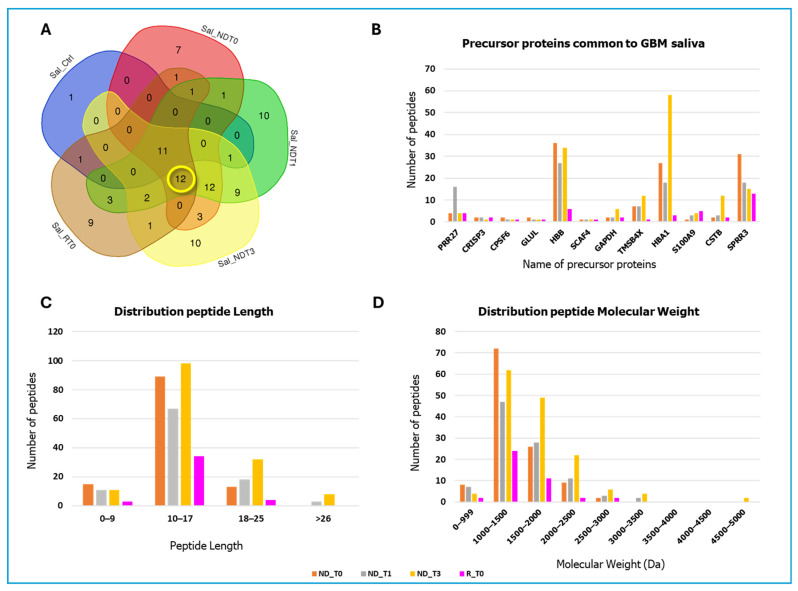
Data on peptides and corresponding precursor proteins obtained by LC-MS analysis of NDT0, NDT1, NDT3, and RT0 GBM saliva pools. (**A**) Venn diagram resulting from grouping analysis of the protein precursors of the peptides identified in pathological saliva pools, highlighting 12 precursor proteins common to all GBM saliva samples, marked in yellow. (**B**) Bar graph illustrates the number of unique peptides identified for each of the 12 shared precursor proteins with quantitative differences highlighted across the analyzed groups: NDT0 (orange), NDT1 (grey), NDT3 (yellow), and RT0 (pink). (**C**) Bar graph illustrates the distribution of the peptide lengths. (**D**) Bar graph illustrating the distribution of molecular weights (Da).

The Venn diagram analysis revealed 12 precursor proteins that were consistently identified in all GBM saliva pools (Figure 5A), but not in CTRL. These proteins include Proline-rich protein 27 (PRR27), Cysteine-rich secretory protein 3 (CRISP3), Cleavage and polyadenylation specificity factor subunit 6 (CPSF6), Glutamine synthetase (GLUL), Hemoglobin subunit beta (HBB), SR-related and CTD-associated factor 4 (SCAF4), Glyceraldehyde-3-phosphate dehydrogenase (GAPDH), Thymosin beta-4 (TMSB4X), Hemoglobin subunit alpha (HBA1), Protein S100A9 (S100A9), Cystatin-B (CSTB), and Small proline-rich protein 3 (SPRR3). In particular, Table 1 lists their peptide fragments, which were identified in all GBM pools, thus marking the GBM tumor independently from tumor type and time of collection.

HBA1 and HBB were found to be particularly abundant in the GBM salivary samples, as highlighted in Figure 5B, where the relatively high counts of their fragment peptides underscore their relevance in this pathology. According to the Human Protein Atlas database [27], HBA1 and HBB are both classified as “genes with elevated expression in GBM,” which could be in accordance with our data in saliva. Furthermore, as reported in Table 1, some peptide fragments from HBA1 (1044.612 *m*/*z* MH^+^) and HBB (1209.648 *m*/*z* MH^+^, 1115.568 *m*/*z* MH^+^, 1214.524 *m*/*z* MH^+^) were identified as common across all pathological salivary samples, highlighting their consistent presence and significance in the GBM-associated samples. These findings suggest that HBA1 and HBB, along with their fragment peptides, may play a crucial role in the molecular mechanisms driving the development and progression of GBM tumors. Particular attention should also be given to S100A9. This protein is classified in the Human Protein Atlas database [27] as “genes associated with unfavorable prognosis GBM”, and it is recognized as a “cancer-related” protein. Moreover, Figure 5B shows a gradual increase in the number of S100A9 peptide fragments identified in RT0 saliva with respect to NDT0, suggesting its potential involvement in the more advanced or aggressive stages of the disease. This increase may indicate an active role of specific fragments of S100A9 in tumor progression and aggressiveness of GBM [30]. As shown in Table 1, the S100A9 peptide fragment with a molecular mass of 1543.692 *m*/*z* MH^+^ was identified as common to all GBM salivary samples, confirming its potential relevance.

Similarly to the data in Figure 2, the lengths of the peptides identified exclusively in GBM pools were largely between 10 and 17 amino acids (Figure 5C), while the molecular mass of the majority of the peptides ranged from 1000 to 2500 Da (Figure 5D). It is noteworthy that the SPPR3 protein exhibited a higher number of peptides identified in the NDT0 compared to the other samples analyzed. It has been demonstrated that this protein plays a crucial role in the initiation and progression of various types of cancers, including GBM [31].

The 12 precursor proteins common to all GBM pools may be of particular interest, since their peptide fragments identified could reflect the presence of a tumor, whether primary or recurrent. These proteins appear to be minimally influenced by temporal variations or therapeutic interventions, such as surgery, chemotherapy, or radiotherapy.

The 18 shared peptides listed in Table 1 are of particular significance. Their consistent presence in all GBM pools and their absence in CTRL could identify a specific disease phenotype or promising targets for further investigation, should they prove to be bioactive peptides.

**Figure 6:**
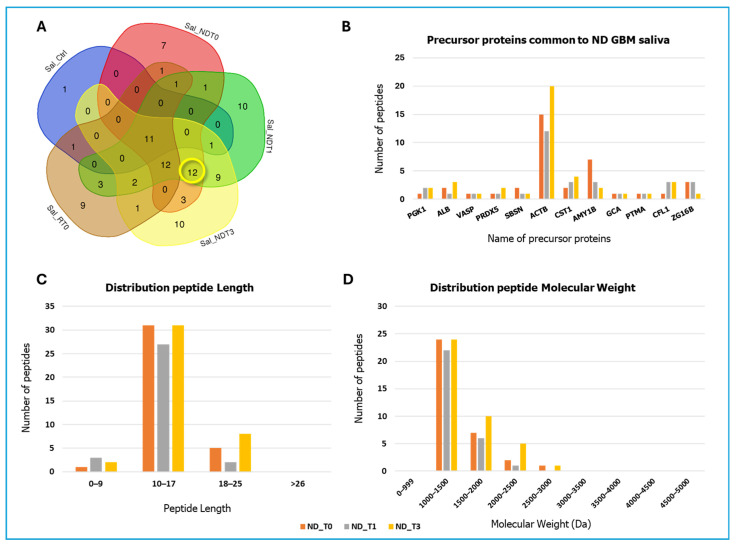
Data on peptides and corresponding protein precursors obtained by LC-MS analysis of NDT0, NDT1, NDT3, RT0 GBM, and CTRL saliva pools. (**A**) Venn diagram resulting from grouping analysis of the protein precursors of the peptides identified in all pools, highlighting 12 precursor proteins common to all ND GBM saliva samples, marked in yellow. (**B**) Bar graph illustrates the number of unique peptides identified for each of the 12 shared precursor proteins, with quantitative differences highlighted across the analyzed groups: NDT0 (orange), NDT1 (grey), and NDT3 (yellow). (**C**) Bar graph illustrates the distribution of the peptide lengths. (**D**) Bar graph illustrates the distribution of molecular weights (Da).

The Venn diagram analysis additionally revealed other different 12 precursor proteins that exclusively mark ND GBM pools (Figure 6A), namely, Phosphoglycerate kinase 1 (PGK1), Albumin (ALB), Vasodilator-stimulated phosphoprotein (VASP), Peroxiredoxin-5, mitochondrial (PRDX5), Suprabasin (SBSN), Actin, cytoplasmic 1 (ACTB), Cystatin SN (CST1), Alpha-amylase 1B (AMY1B), Grancalcin (GCA), Prothymosin alpha (PTMA), Cofilin-1 (CFL1), and Pancreatic adenocarcinoma upregulated factor (ZG16B). Table 2 lists the relative peptide sequences commonly identified in all ND GBM saliva pools. The histogram in Figure 6B shows for this group of proteins a more similar number of fragment peptides identified, with the exception of ACTB, CST1, and AMY1B, resulting in the main contributors to the set of peptides identified. Particularly, the ACTB (beta-actin), which is a protein upregulated in gliomas and particularly in malignant gliomas [32], showed the highest number of identified peptides in the group, six out of them common to all ND GBM pools. This protein precursor, recognized in all tumor cells [32], plays a crucial role in cell motility and division, as well as in immune response and cellular transformation.

Among the other peptides identified, of note in Table 2 is the small albumin fragment 423–431 GEYKFQNAL (1069.529 *m*/*z* MH^+^), identified as a urinary biomarker for the differential diagnosis of vasculitis [33] and highlighted for potential clinical application in diagnostics.

The fragment 2-15 SDAAVDTSSEITTK of Prothymosin alpha (PTMA), which is identified in all ND GBM pools (Table 2), is the N-terminal sequence of the peptide fragment of PTMA 2–29, known as thymosin alpha-1. The peptide SDAAVDTSSEITTK was included in the list of peptide fragments of PTMA identified in tissues of head–neck cancer and oral premalignant lesions [34]. Furthermore, the expression of PTMA has been observed in gliomas across various grades, with overexpression being particularly evident in the higher-grade tumors, as reported by Kumar et al. [35]. This finding aligns with data from the Human Protein Atlas [27], which associates PTMA gene expression with unfavorable prognosis in GBM. Interestingly, the number of peptides identified for PTMA remains constant across the ND GBM pools (Figure 6B). The lengths of the peptide fragments of the 12 precursor proteins were principally between 10 and 17 amino acid residues (Figure 6C), while their molecular weight ranged predominantly between 1000 and 2000 Da (Figure 6D).

Although no shared peptide fragments of ZG16B were identified, this precursor, found exclusively in GBM ND samples, is notable for its reported role as a biomarker in several malignancies, including pancreatic, colorectal, cervical, prostate, oral, ovarian, and breast cancers [36,37,38,39,40,41,42,43]. Table 3 lists the sequences and saliva pools for the identification of their relative peptide fragments.

**Figure 7:**
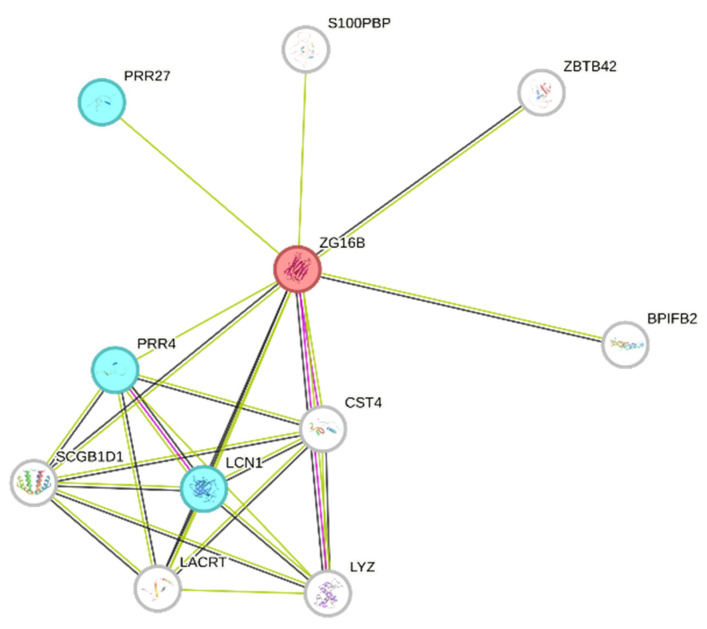
The protein interaction network of ZG16B (red node) was generated using STRING tool [44]. Light blue nodes indicate proteins identified in GBM saliva, and white nodes indicate ZG16B interactors. The edges represent protein–protein relationships, with each color indicating a specific type: known interactions (light blue: curated databases, purple: experimentally determined); predicted interactions (green: gene neighborhood, red: gene fusions, blue: gene co-occurrence); others (ochre: text mining, black: co-expression, light blue: protein homology).

Interestingly, ZG16B showed functional interactions with other precursor proteins identified in the GBM saliva pools (STRING network of Figure 7), such as PRR4, PRR27, and LCN1 (light blue nodes). PRR4 was the unique precursor protein of the group of predicted ZG16B interactors also identified in CTRL saliva.

**Figure 8:**
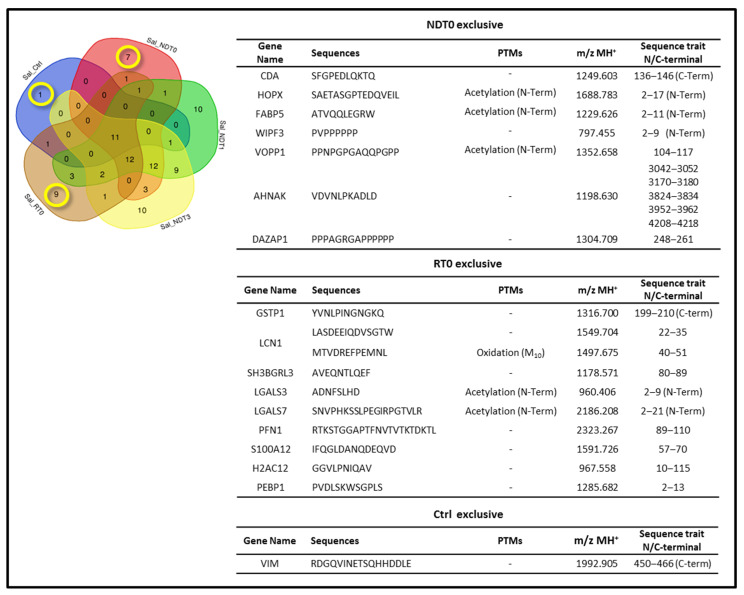
Data on peptides and corresponding precursor proteins obtained by LC-MS analysis of NDT0, NDT1, NDT3, RT0 GBM, and CTRL saliva pools. The Venn diagram resulting from grouping analysis of the precursor proteins of the peptides identified highlights precursor proteins exclusive to ND and R T0 saliva, and CTRL saliva, all marked in yellow. The tables show the details of the peptides exclusively identified in NDT0, RT0, and CTRL saliva.

To further investigate the distinct peptide profile of GBM at different stages, we focused on the peptides identified as exclusive to ND and R GBM saliva samples, specifically at T0 (highlighted in yellow in the Venn diagram of Figure 8). The peptide sequences identified as unique to ND and R GBM T0 saliva are listed in Figure 8. The seven peptides exclusive to saliva NDT0 are fragments of seven proteins, namely, Cytidine deaminase (CDA), Homeodomain-only protein (HOPX), Fatty acid-binding protein 5 (FABP5), WAS/WASL-interacting protein family member 3 (WIPF3), WW domain-binding protein VOPP1 (VOPP1), Neuroblast differentiation-associated protein AHNAK (AHNAK), and DAZ-associated protein 1 (DAZAP1).

A particular focus was placed on three different peptide fragments, namely, the fragment of HOPX (1688.783 *m*/*z* MH^+^), VOPP1 (1352.658 *m*/*z* MH+), and FABP5 (1229.626 *m*/*z* MH+), exclusive of NDT0 saliva. According to the Human Protein Atlas database [27], these proteins are highly expressed in gliomas. VOPP1, also known as Glioblastoma Amplified and Secreted Protein or EGFR-Co-amplified and Over-expressed Protein, is known to be overexpressed in multiple tumor types [45,46,47], including GBM [43], supporting its potential role in tumor progression. HOPX is a protein acting as an oncogene; however, the precise function and tissue distribution of the different transcripts have not yet been described [48]. In the specific case of glioblastomas, HOPX is largely unexpressed, and its regulation contributes to the development of the disease [49,50]. FABP5 is associated with genes linked to unfavorable prognosis in GBM, as reported by the Human Protein Atlas database. Upregulated expression of FABP5 has been observed in different tumor tissues, including cholangiocarcinoma, esophageal carcinoma, GBM, head and neck squamous cell carcinoma, kidney renal clear cell and papillary carcinoma, prostate adenocarcinoma, endometrial carcinoma of the corpus uteri [51], as well as in GBM CUSA fluid and saliva in our previous investigations [18,21]. Therefore, identification of VOPP1, HOPX, and FABP5 fragment peptides in GBM saliva could be in accordance with the literature data, although the specific role of these peptides, if any, is unknown.

The ten peptide sequences exclusive to saliva RT0 (Figure 8) originate from the following nine precursor proteins: Glutathione S-transferase P (GSTP1), Lipocalin-1 (LCN1), SH3 domain-binding glutamic acid-rich-like protein 3 (SH3BGRL3), Galectin-3 (LGALS3), Galectin-7 (LGALS7), Profilin-1 (PFN1), Protein S100A12 (S100A12), Histone H2A type 1-H (H1-2), and Phosphatidyl–ethanolamine-binding protein 1 (PEBP1). It is worth mentioning that the PFN1 peptide RTKSTGGAPTFNVTVTKTDKTL (2323.267 *m*/*z* MH^+^) was identified in medulloblastoma DAOY cells by top–down LC-ESI-Orbitrap Elite MS/MS proteomic analysis in our previous investigation [52].

It is finally of note that only one Uniprot accession was exclusively identified in CTRL saliva, namely, Vimentin protein (VIM), through detection of its C-terminal fragment 450-466 with sequence RDGQVINETSQHHDDLE (1992.905 *m*/*z*, MH^+^) (Figure 8). Although VIM is predominantly cytoplasmic, the protein is also secreted and/or present in the cells’ extracellular surface by exerting different roles [53]. VIM has already been identified in saliva in relation to oral cancer [54] and SARS-CoV-2 infection [55]. Interestingly, VIM undergoes cleavage by caspases during apoptosis, generating several fragment products. This proteolytic process plays an important role in the intermediate filament disassembly and promotion of apoptosis, recognized for its pro-apoptotic N-terminal product [56]. Therefore, the absence of the C-terminal fragment of VIM in the tumor saliva could mark the absence of VIM fragmentation in some way connected to the promotion of apoptosis, a process that is impaired in cancer. In a very recent paper investigating VIM fragmentation in relation to Alzheimer’s disease [57], VIM was discovered as a substrate of Asparagine endopeptidase (AEP/δ-secretase), producing its complementary N- and C-terminal fragments 1–283 and 284–466, respectively, both exhibiting, but especially the C-terminal, a pro-apoptotic action. Although this proteolytic process of VIM is pathological, the recognition of a pro-apoptotic role also for the C-terminal part of the protein is interesting.

**Figure 9:**
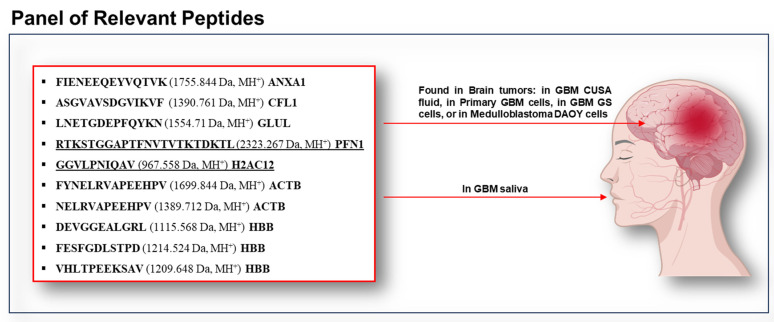
Peptide sequences identified in GBM saliva (top left), as well as in different brain tumor specimens (listed on the right), namely, GBM CUSA fluid [20], primary GBM cells and GS GBM cells [65], and medulloblastoma DAOY cells [52]. These peptides emerge as potential panel of salivary biomarkers for the diagnosis and monitoring of GBM tumors. The two underlined fragments could mark GBM recurrence because they are exclusively identified in RT0 GBM saliva. The image was processed using BioGDP generic Diagramming Platform for Biomedical Graphics [66].

The proteomic characterization of the low molecular mass proteome/peptidome of the acid-soluble fraction of GBM saliva revealed a set of peptides with potential relevance to GBM disease. Among these are included either the peptides identified in all pools showing statistically significantly different levels, or the peptides exclusively identified in ND or R GBM, or in both of them. It is noteworthy that some of them originated from the fragmentation of proteins known to be highly expressed in GBM and/or associated with poor prognosis. Based on data from the literature and our previous investigations on GBM tumor CUSA aspirate fluid [20], notably, a subset of 10 peptides (Figure 9) among the total identified resulted in particular interest. In fact, their co-identification in GBM saliva, CUSA fluid, and tumor cells is a data match that could support their potential role in the disease.

The fragment 13-26 FIENEEQEYVQTVK (1755.844 *m*/*z* MH^+^) of ANXA1 (Figure 9), identified in NDT0, T1, and RT0 GBM saliva, has also been identified in GBM CUSA aspirate fluid from ND and R GBM in our previous study [20]. This peptide corresponds to the end part of the N-terminal peptide 2–26 of ANX1, called Annexin Ac2-26, cleaved by cathepsin G and exhibiting anti-inflammatory, neuro- and cardioprotective, and wound-healing biological activities [58,59,60,61], but also a role in microglia [62], involved in GBM onset and diffusion [63] and in alternative macrophage polarization in tumor microenvironment [64].

Similarly, the N-terminal fragment ASGVAVSDGVIKVF (1390.761 *m*/*z* MH^+^) of CFL1 (Figure 6 and Figure 9) was identified in the saliva of ND GBM patients (NDT0, T1, and T3). Interestingly, this fragment has been previously detected in ND GBM CUSA fluid of the CORE zone and tumor periphery [20], but not in R GBM samples, suggesting its potential biomarker role in the early stages of tumor development.

The C-terminal fragment LNETGDEPFQYKN (1554.71 *m*/*z* MH^+^) from GLUL (Figure 5 and Table 1) was identified in all GBM saliva pools (NDT0, T1, T3, and RT0) in the present investigation and consistently in ND and R GBM CUSA fluid in our previous investigation [20]. Interestingly, this peptide fragment was identified as an HLA-II antigen in glioblastoma stem-like cells (GS9) [65]. Further investigations would be interesting to determine what role the peptide could have in the disease.

The fragment GGVLPNIQAV (967.558 *m*/*z* MH^+^) of H2AC12 (Figure 8 and Figure 9) was exclusively detected in the RT0 GBM saliva and identified as an HLA-II antigen in glioblastoma stem-like cells (GS9) [65]. Likewise, the PFN1 fragment RTKSTGGAPTFNVTVTKTDKTL (2323.267 *m*/*z* MH^+^) (Figure 8 and Figure 9) was found exclusively in the saliva of RT0 patients and identified in DAOY medulloblastoma cells [52].

Two fragments of ACTB, FYNELRVAPEEHPV (1699.844 *m*/*z* MH^+^), and NELRVAPEEHPV (1389.712 *m*/*z* MH^+^) (Figure 6 and Figure 9) were exclusively identified in ND GBM saliva, all pools. The first was identified in glioblastoma stem-like cells (GS9), while the second was also found in primary GBM cells (GBMZH466) and multiple GS cell lines (GS2, GS5, GS9) [65].

Three fragments of HBB, namely, the peptides HLTPEEKSAV (1209.648 Da MH^+^), DEVGGEALGRL (1115.568 *m*/*z* MH^+^), and FESFGDLSTPD (1214.524 Da MH^+^), were consistently found in the saliva of all GBM patients (NDT0, T1, T3, and RT0). The protease potentially responsible for the generation of the HBB peptide DEVGGEALGRL (1115.568 *m*/*z* MH^+^) is cathepsin D (CTSD), following the MEROPS database (https://www.ebi.ac.uk/merops, accessed on 13 May 2025), an enzyme highly expressed in GBM and strongly associated with radioresistance [67]. This fragment was also identified in primary glioblastoma cells (GBMZH483, GBMZH446, GBMZH450, GBMZH457, GBMZH466) and GS cells (GS2, GS5, GS9) [65]. Similarly, DEVGGEALGRL (1115.568 *m*/*z* MH^+^) was detected in primary glioblastoma cells (GBMZH446, GBMZH483, and GBMZH480), GS cells (GS5, GS9), as well as both the CUSA fluid from the CORE zone and tumor periphery of the GBM tumor relapse [20]. The third fragment (1214.524 *m*/*z* MH^+^) was identified in primary glioblastoma cells (GBMZH466, GBMZH483) [65].

The detection of specific peptide fragments in GBM samples suggests that proteases play an active role in the selective cleavage of proteins within the tumor microenvironment, potentially impacting key signaling pathways and metabolic adaptations. Among these, cathepsins, and particularly cathepsin B, D, and G, are well known for their involvement in GBM progression, facilitating tumor cell migration, invasion, and resistance to therapy [68,69]. Notably, our previous study identified these and other proteases in GBM saliva after trypsin digestion [21], further supporting the present findings.

## 3. Materials and Methods

### 3.1. Sample Collection

Pathological saliva samples were collected from three patients (median age 52.3 ± 6.43) affected by newly diagnosed (ND) GBM and from three patients with relapsed (R) GBM (mean age 48.6 ± 8.1 years) (Table S6) after written consent. This study was approved by the Ethical Committee of Catholic University of Rome with number 13891/18 ID 2015, date of approval 18 May 2018. Control saliva samples (CTRL) were collected from three healthy volunteers (mean age 52.3 ± 4.6 years). Saliva samples (approximately 0.3 mL) were collected pre-surgery (T0 saliva) and during the post-surgery follow-up, namely, 1-month and 3-month saliva (T1 and T3, respectively) for ND GBM, the latter after radio- and temozolomide chemotherapy-combined treatment. For R GBM, only T0 saliva was collected. Whole saliva samples were collected between 11:00 and 12:00 am in the fasting state and without stimulation, after rinsing the oral cavity. Whole saliva was collected as it flowed into the anterior floor of the mouth with a soft plastic pipette for less than 1 min, transferred to a plastic tube, and treated within 30 min from collection by addition of 0.2% formic acid (FA) (LC-MS grade, Merck, Darmstadt, Germany). aqueous solution (*v*/*v*) in a 1:1 volume ratio, followed by centrifugation (4 °C, 24,000× *g* 5 min). The resulting supernatant acid-soluble fraction was collected and stored at −80 °C until proteomic analysis.

### 3.2. Chemicals

Protease Inhibitor Cocktail (PIC) (AEBSF, aprotinin, bestatin, E-64, EDTA, and leupeptin) was from Sigma-Aldrich (St. Louis, MO, USA). Water, all LC-MS grade, was from Merck (Darmstadt, Germany). FASP (Filter- Aided Sample Preparation) centrifugal filter units Ultracel PL-10, regenerated cellulose 10,000 NMWL, Microcon ^®^-10 were from Merck Millipore Ltd. (Tullagree, Carrigtwohill, Co Cork, Irland).

### 3.3. Sample Preparation

The saliva samples’ acid-soluble fractions were pooled according to tumor type, ND and R GBM, and time of collection (T0, T1, and T3), and pretreated using FASP (Filter-Aided Sample Preparation) centrifugal filter units with a molecular cutoff of 10 kDa, following the procedure previously described in [70]. Specifically, a volume of each saliva pool corresponding to 50 or 30 µg of total protein content was mixed with 0.1% (*v*/*v*) aqueous FA solution up to a final volume of 200 μL and loaded in the FASP centrifugal filter unit. After centrifugation at 11,000× *g* for 15 min at 4 °C, two proteome fractions were recovered. The proteome fraction > 10 kDa, retained by the filter, underwent trypsin digestion and was analyzed by LC-MS by a bottom–up proteomic approach in our previous investigation [21]. The complementary low molecular mass fraction of the proteome < 10 kDa, unretained by the filter, was collected unchanged, added with protease inhibitor cocktail (PIC, 10× concentrated solution), lyophilized, and stored at −80 °C until LC-MS analysis in intact form (top–down approach), as the object of the present investigation. Prior to the LC-MS analysis, the samples were thawed and redissolved in 0.1% (*v*/*v*) FA aqueous solution in order to be injected into the chromatographic column, with a total protein content ranging from 1 to 5 μg.

### 3.4. LC-MS Analyses

LC-MS analyses of samples were performed in triplicate using the UltiMate 3000 RSLCnano System coupled to an Orbitrap Elite MS detector with EASY-Spray nanoESI source (Thermo Fisher Scientific, Waltham, MA, USA) and Thermo Xcalibur 2.2 computer program (Thermo Fisher Scientific, Waltham, MA, USA) for instrumental operation and data acquisition. Chromatographic separations were performed on EASY-Spray C18 columns (15 cm × 50 µm I.D., 2 µm particles, 100 Å pore size) (Thermo Fisher Scientific, Waltham, MA, USA) in coupling with PepMap100 nano-trap cartridge (C18, 5 µm, 100 Å, 300 µm i.d. × 5 mm) (Thermo Fisher Scientific, Waltham, MA, USA). Separation was performed at 40 °C in gradient elution, at mobile phase flow rate of 0.3 μL/min, using aqueous FA solution (0.1%, *v*/*v*) as eluent A and ACN/FA solution (99.9:0.1, *v*/*v*) as eluent B as follows: (i) 5% B (7 min), (ii) from 5% to 35% B (113 min), (iii) from 35% B to 99% (2 min), (iv) 99% B (3 min), (v) from 99% to 1.6% B (2 min), (vi) 1.6% B (3 min), (vii) from 1.6% to 78% B (3 min), (viii) 78% B (3 min), (ix) from 78% to 1.6% B (3 min), (x) 1.6% B (3 min), (xi) from 1.6% to 78% B (3 min), (xii) 78% B (3 min), (xiii) from 78% B to 5% B (2 min), (xiv) 5% B (20 min). The injection volume was 5 μL. The Orbitrap Elite instrument was operating in positive ionization mode at a 60,000 full scan resolution in 350–2000 *m*/*z* acquisition range, performing MS/MS fragmentation by collision-induced dissociation (CID, 35% normalized collision energy) of the 20 most intense signals of each MS spectrum in Data-Dependent Scan (DDS) mode. The minimum signal was set to 500.0, the isolation width to 2 *m*/*z,* and the default charge state to +2. MS/MS spectra acquisition was performed at a resolution of 60,000 and with an isolation width of 5 *m*/*z*. CTRL saliva pool analyses were performed by UHPLC-MS/MS UltiMate 3000 RSLCnano System coupled to Orbitrap Fusion Lumos Tribrid Mass Spectrometer and EASY-Spray nanoESI (Thermo Fisher Scientific, Waltham, MA, USA) following the same chromatographic and mass spectrometer operating conditions as described above.

### 3.5. Data and Bioinformatics Analysis

LC-MS and MS/MS data were elaborated by Proteome Discoverer software (version 1.4.1.14, Thermo Fisher Scientific, Waltham, MA, USA), based on SEQUEST HT cluster as search engine against the Swiss-Prot Homo Sapiens proteome (UniProtKb, Swiss-Prot, homo+sapiens released in January 2024) by applying the following spectrum filters: minimum precursor mass 350 Da, maximum precursor mass 10,000 Da, total intensity threshold 0.0, and minimum peak count 1. The signal-to-noise (S/N) threshold was set to 1.5. “No enzyme” was set for top–down analyses; minimum and maximum peptide lengths were 6 and 144 residues, respectively. Mass tolerance was set at 10 ppm; fragment mass tolerance was set at 0.02 Da; use average precursor mass was set as False; use average fragment mass was set as False. The set dynamic modifications were methionine oxidation (+15.99 Da) and N-Terminus acetylation (+42.011 Da). The identifications were validated by the Percolator node, with the strict target value of False Discovery Rate (FDR) set at 0.01 and the relaxed value at 0.05. Identification data were filtered for high confidence and peptide rank 1. Data were further filtered to exclusively select the identifications of unique peptides obtained with high confidence in all three analytical replicates per sample, as applied in our previous peptidomics investigation on GBM CUSA fluid [20]. The Venn diagram tool [26] was used to highlight common and selective elements of the different analyzed pools. Gene Ontology (GO) analysis was performed by Protein ANalysis THrough Evolutionary Relationships (PANTHER) [71], while the STRING tool version 12.0 was used to predict protein–protein functional interactions [44]. The selected classification of proteins was performed using the “cancer-related proteins”, “genes are associated with unfavorable prognosis GBM”, “genes are associated with favorable prognosis GBM”, “genes elevated expression in GBM”, “gene only detected in glioblastoma multiforme”, and “genes elevated expression in salivary gland” subclass list of proteins available on the Human Protein Atlas database [27] (accessed on January 2025).

Relative quantitative analysis was performed by a label-free approach using peptides’ area values resulting from Proteome Discoverer LC-MS data elaboration, considering the average peptide area values of the three analytical replicates. One-way ANOVA with Turkey’s post hoc test (GraphPad Prism9 software) was applied for statistical evaluation, considering *p*-values < 0.05 as significant.

## 4. Conclusions

The analysis of the salivary peptidome in GBM patients remains an underexplored field of research. In recent years, saliva has attracted increasing attention as a promising source of biomarkers, offering unique opportunities compared to other biofluids [10,72]. Its low-invasive and high patient compliance collection, along with its rich molecular composition and low presence of interfering abundant proteins, make saliva an ideal biofluid for investigations on large-scale and clinical applications [73].

This study presents, for the first time, the proteomic profiling of the low molecular fraction < 10 kDa of the acid-soluble fraction of ND and R GBM saliva in comparison to CTRL by a top–down LC-MS approach to identify small proteins and peptides in their intact form, aiming at identifying significant variations that could be valuable for future investigations on a large scale. This pilot study, conducted on a limited number of pooled salivary samples, aimed to assess the feasibility of salivary peptidomic profiling as a source of potential GBM biomarkers. Specifically, 11 precursor proteins, namely, PRR4, HTN1, STATH, MUC7, PRH1, SMR3B, PRB1-2-3-4, and PIGR, consistently present across all saliva pools analyzed, showed variations in the profile of their fragment peptides identified. These variations include differences in peptide number, length, and molecular mass, evidencing variable proteolytic events occurring among the pools, which could reflect different protein degradation processes or protease activities that feature GBM tumor [74,75].

Differently, 12 precursor proteins, namely, PRR27, CRISP3, CPSF6, GLUL, HBB, SCAF4, GAPDH, TMSB4X, HBA1, S100A9, CSTB, and SPRR3, distinguished GBM saliva from CTRL, being commonly identified in both ND and R GBM T0 pre-surgery saliva. Other different 12 precursor proteins, namely, PGK1, ALB, VASP, PRDX5, SBSN, ACTB, CST1, AMY1B, GCA, PTMA, CFL1, and ZG16B, marked all ND GBM saliva pools, highlighting key peptides potentially linked to tumor onset and progression. Notably, the peptide fragment of S100A9, detected in GBM saliva but not in CTRL, could confirm the outlined role of S100A9 in tumor progression and aggressiveness [30]. Additionally, the fragment peptides of HBA1, HBB, GLUL, ACTB, and CFL1 in Figure 9, exclusively marking GBM saliva and also identified in GBM tumor cells [65] and GBM CUSA fluid [20], could represent a potential panel of GBM biomarkers to further investigate.

Particularly relevant was the identification of peptides exclusive of ND or R pre-surgery saliva, which could disclose potential diagnostic biomarkers of tumor onset or tumor relapse with potential application in post-surgery follow-up. ND GBM pre-surgery saliva (NDT0) showed the exclusive characterization of peptide fragments of CDA, HOPX, FABP5, WIPF3, VOPP1, AHNAK, and DAZAP1 proteins, which, to the best of our knowledge, have not been previously identified in relation to GBM tumor. R GBM pre-surgery saliva (RT0) showed the exclusive identification of peptide fragments of GSTP1, LCN1, SH3BGRL3, LGALS3, LGALS7, PFN1, S100A12, H2AC12, and PEBP1 proteins. All these peptides, marking one or another saliva pool, may represent distinctive biomarkers of different GBM stages or conditions, suggesting their potential diagnostic or prognostic values. Furthermore, peptides common to both ND and R T0 saliva could depict the consistent peptidomic profile of the tumor independently of disease state, namely, the H1-2 fragments and the fragment of ANXA1, the latter shared in addition by NDT1 saliva.

This investigation, performed on salivary pools on a small scale, does not intend to draw conclusions that would not be supported by adequate statistics, but to investigate the prerequisites for a larger-scale study of saliva in GBM, favored by the reduced invasiveness and good patient compliance of the biofluid, for potential future development of clinical applications in the direction of precision medicine. In summary, our results highlight the promising role of saliva biofluid as a source of potential GBM biomarkers, complementing our previous investigation by a bottom–up approach [21] and adding interesting new hints. This pilot study opens new perspectives in the understanding of the peptidomic profile of GBM through saliva, suggesting a panel of candidate peptide biomarkers, including peptide fragments of ANXA1, CFL1, GLUL, PFN1, H2AC12, ACTB, and HBB, which, having already been identified in other GBM samples and cell lines, warrant further exploration and validation on large scale with the aim of promoting progress in precision medicine, improving diagnostic and prognostic capabilities in the clinical setting.

## Figures and Tables

**Table 1:** Selected list of the peptide sequences and relative protein precursors commonly identified in all GBM saliva pools.

Gene Name	Peptide Sequence	PTMs	*m* **/*z* MH^+^**	Peptide Length	Sequence Position N/C-Terminal	Sequence Trait
PRR27	FIGEDDNDDGHPLHPS	-	1764.745	16	-	22–37
	KRRFPFIGEDDNDDGHPLHPS	-	2449.160	21	-	17–37
CRISP3	NEDKDPAFTAL	-	1220.580	11	-	21–31
CPSF6	PQQGPPPPPG	-	971.494	10	-	312–321
GLUL	LNETGDEPFQYKN	-	1554.709	13	C-term	361–373
HBB	VHLTPEEKSAV	-	1209.649	11	N-term	2–12
	DEVGGEALGRL	-	1115.568	11	-	22–32
	FESFGDLSTPD	-	1214.524	11	-	43–53
SCAF4	FGPGVPPPPPPP	-	1155.618	12	-	712–722
TMSB4X	SDKPDMAEIEKF	Acetylation (N-term) Oxidation (M6)	1451.675	12	N-term	2–13
HBA1	VDPVNFKLL	-	1044.612	9	-	94–102
S100A9	DTNADKQLSFEEF	-	1543.692	13	-	67–79
SPRR3	KQTFTPPPQLQQQ	-	1540.810	13	-	7–20
	TFTPPPQLQQQ	-	1284.659	11	-	9–20
	TFTPPPQLQ	-	1028.541	9	-	9–17
	VKQPSQPPPQEIFVPT	-	1791.966	16	-	21–36
	AIKVPEQGYT	-	1105.589	10	-	130–139
	FTPPPQLQ	-	927.492	8	-	10–17

**Table 2:** Selected list of the peptide sequences and related protein precursors commonly identified in all ND GBM saliva pools.

Gene Name	Peptide Sequence	PTMs	*m*/*z* MH^+^	Peptide Length	Sequence Position N/C-Terminal	Sequence Trait
ALB	GEYKFQNAL	-	1069.529	9	-	423–431
VASP	GPPAPPAGGPPPPP	-	1205.631	14	-	161–174
PRDX5	APIKVGDAIPAVEV	-	1378.795	14	-	54–67
SBSN	ASDDPIEKVIE	-	1215.611	11	-	24–34
ACTB	FYNELRVAPEEHPV	-	1699.844	14	-	90–103
	LLTEAPLNPKAN	-	1280.723	12	-	104–115
	GFAGDDAPRAVFPS	-	1406.668	14	-	20–33
	VAPEEHPVLLTEAPLNPK	-	1954.066	18	-	96–113
	FAGDDAPRAVFPS	-	1349.651	13	-	21–33
	NELRVAPEEHPV	-	1389.713	12	-	92–103
GCA	AYPGYGGGFGNF	Acetylation (N-term)	1248.532	12	N-term	2–13
PTMA	SDAAVDTSSEITTK	Acetylation (N-term)	1466.685	14	N-term	2–15
CFL1	ASGVAVSDGVIKVF	Acetylation (N-term)	1390.759	14	N-term	2–15

**Table 3:** List of the ZG16B peptide fragments identified in ND GBM (T0, T1, and T3 saliva samples).

Gene Name (Sample Pool of Identification)	Peptide Sequence	PTMs	*m*/*z* MH^+^	Peptide Length	Sequence Position N/C-Terminal	Sequence Trait
ZG16B (NDT0)	GPGGGKYFSTTEDY	-	1478.641	14	-	21–34
	KLGALGGNTQEV	-	1186.641	12	-	64–75
	FEWNYPLEEPT	-	1424.638	11	-	145–155
	GDSWDVKLG	-	976.472	9	-	58–66
ZG16B (NDT1)	TLQPGEYITKVF	-	1395.753	12	-	76–87
	EDYDHEITGL	-	1191.514	10	-	32–41
ZG16B (NDT3)	TLQPGEYITKVF	-	1395.753	12	-	76–87

## Data Availability

Data is contained within the article or Supplementary Materials.

## Supplementary Materials

The following supporting information can be downloaded at https://www.mdpi.com/article/10.3390/ijms26209995/s1.

## Abbreviations

The following abbreviations are used in this manuscript:

GBM	Glioblastoma IDH wild type
ND GBM	Newly Diagnosed GBM
R GBM	Recurrent GBM
T0	Pre-surgery saliva
T1	1-month post-surgery saliva
T3	3-month post-surgery saliva
CUSA	Cavitron Ultrasonic Surgical Aspirator
FASP	Filter-Aided Sample Preparation
LC	Liquid Chromatography
MS	Mass Spectrometry
CNS	Central Nervous System
FA	Formic acid
ACN	Acetonitrile
PIC	Protease inhibitor cocktail
FDR	False Discovery Rate
