# Supplementary material for: Proteomic Profiling of Pre- and Post-Surgery Saliva of Glioblastoma Patients II: A Preliminary Investigation of the Complementary Low Molecular Mass Fraction
Source: Int J Mol Sci. 2025 Oct 14;26(20):9995. doi: 10.3390/ijms26209995 (PMC12562582; doi:10.3390/ijms26209995)
Supplement: Supplementary file 1 [file ijms-26-09995-s001.zip › ijms-3892957-supplementary/Table S6.pdf]

Table S6. Saliva sample pools specifications and GBM patients' data.

**Patient ID**	**age** (year)	**gender** M, F	**GBM** ND=Newly Diagnosed R=Relapse	**lobe of the lesion** temporal =1 parietal =2 occipital =3	**lesion side** left=L right=R	**IDH-1** Wt=wild type M= mutated	**Saliva collected samples**		
							**T0**	**T1**	**T3**
**ND GBM saliva pool***									
**PP1**	57	M	ND	2/3	L	Wt	x	x	x
**PP12**	55	M	ND	1	R	Wt	x	x	x
**PP14**	45	F	ND	2	R	Wt	x	x	x
**R GBM saliva pool***									
**PP4**	50	M	R	1	L	Wt	x	x	x
**PP5**	56	M	R	2	R	Wt	x	x	x
**PP6**	40	F	R	2	R	Wt	x	x	x

*Saliva was collected pre-surgery (T0 saliva) for ND and R GBM, and 1 months and 3 months post-surgery follow-up (T1 and T3, respectively) for ND GBM. T3 saliva was collected after radio- and temozolomide chemotherapy combined treatment.
